# The genome sequence of *Inga* *oerstediana* Benth.

**DOI:** 10.12688/wellcomeopenres.23146.1

**Published:** 2024-10-17

**Authors:** Rowan J. Schley, R. Toby Pennington, Alex D. Twyford, Kyle G. Dexter, Catherine Kidner, Todd P. Michael, Royal Botanic Garden Edinburgh Genome Acquisition Lab, Plant Genome Sizing collective, Wellcome Sanger Institute Tree of Life Management, Samples and Laboratory team, Wellcome Sanger Institute Scientific Operations: Sequencing Operations, Wellcome Sanger Institute Tree of Life Core Informatics team, Tree of Life Core Informatics collective

**Affiliations:** 1 University of Exeter, Exeter, England, UK; 2 Royal Botanic Garden Edinburgh, Edinburgh, Scotland, UK; 3 University of Edinburgh, Edinburgh, Scotland, UK; 4 University of Turin, Turin, Italy; 5 Salk Institute for Biological Studies, La Jolla, California, USA; 6 University of California San Diego, San Diego, California, USA; 7 San Diego Botanical Garden, San Diego, California, USA

**Keywords:** Inga oerstediana, genome sequence, chromosomal, Fabales

## Abstract

We present a genome assembly from an individual of *Inga oerstediana* (Streptophyta; Magnoliopsida; Fabales; Fabaceae). The genome sequence has a total length of 970.60 megabases. Most of the assembly is scaffolded into 13 chromosomal pseudomolecules. The mitochondrial and plastid genome assemblies have lengths of 1,166.81 and 175.18 kilobases, respectively. Gene annotation of this assembly on Ensembl identified 33,334 protein-coding genes.

## Species taxonomy

Eukaryota; Viridiplantae; Streptophyta; Streptophytina; Embryophyta; Tracheophyta; Euphyllophyta; Spermatophyta; Magnoliopsida; Mesangiospermae; eudicotyledons; Gunneridae; Pentapetalae; rosids; fabids; Fabales; Fabaceae; Caesalpinioideae; mimosoid clade; Ingeae; *Inga*; *Inga oerstediana* Benth. (NCBI:txid486073).

## Background

*Inga* Mill. (Fabaceae) is a ubiquitous and characteristic component of the species-rich neotropical rainforest flora, typifying the rapid evolutionary radiations that generated most neotropical tree diversity. Indeed, *Inga* exhibits the highest diversification rate of any Amazonian tree genus ( Baker *et al.*, 2014; Richardson *et al.*, 2001). *Inga oerstediana* Benth. is a widespread tropical rainforest tree species, growing up to 30m tall. This species occurs from southern Mexico through Central America southwards to Bolivia, as well as on the Caribbean islands of Grenada and Trinidad ( Pennington, 1997). Within South America, *Inga oerstediana* can be confused with its sister species *I. edulis*, but they largely segregate geographically. *Inga oerstediana* is found within the Andes and west of the Andes, while *I. edulis* is widespread across the Amazon Basin and elsewhere east of Andes, with the two species ranges overlapping in the eastern foothills of the Andes. *Inga oerstediana* displays broad ecological tolerance, being found from 0–3000m in elevation, and while mostly found in perma-wet rainforest this species also occurs in the seasonally dry climate of Ecuador’s Pacific coast.

Tropical rainforest tree species like *Inga oerstediana* experience high levels of herbivory and have accordingly evolved several means by which to defend themselves. Like all *Inga* species, *I. oerstediana* possesses extra-floral nectaries on its leaf midribs that attract ants for defence against herbivores ( Pennington, 1997), and produces a cocktail of defensive chemicals (including flavan3ol monomers, Forrister *et al.*, 2023) in its young leaves to defend them against herbivory. The wide ecological tolerance and broad, spreading crown of *I. oerstediana* also renders it ideal for use as a shade tree in coffee and cacao cultivation ( Grossman *et al.*, 2006), and it is commonly used for fuel wood ( Pennington, 1997). This species is also widely used for alley-cropping and agroforestry due to its ability to fix nitrogen ( Hands, 1998), while also being cultivated for its edible fruits, which have a sweet, white seed coat (sarcotesta) surrounding the seeds ( Pennington, 1997). As a result, both *Inga oerstediana* and *Inga edulis* are cultivated widely in the tropical Americas. The sample sequenced here, originally from the province of Napo in the Ecuadorian Amazon but grown at RBGE, was diploid (2 *n*=2 *x*=26) as per previous records for the species ( Hanson, 1995).

Here we present one of three chromosomally complete, annotated genome sequences for *Inga,* which are the first for the genus. Specifically, this *Inga oerstediana* genome will be of great utility in future work, given the importance of this species in agroforestry settings. Furthermore, the species-rich genus *Inga* is a well-established study system for understanding the ecology and evolution of tropical rainforest floras, and so the genomic resources we present here will also be of great utility for such work. Potential avenues for future work using this genome may include exploring the genomic underpinnings of this species’ broad ecological tolerance to improve its utilisation in agroforestry, as well as to understand patterns of genetic diversity in cultivated populations of *I. oerstediana*. In addition, this reference genome will be a useful resource for comparative genomic work examining the evolution of defence chemistry across *Inga*.

## Genome sequence report

**Figure 1.**
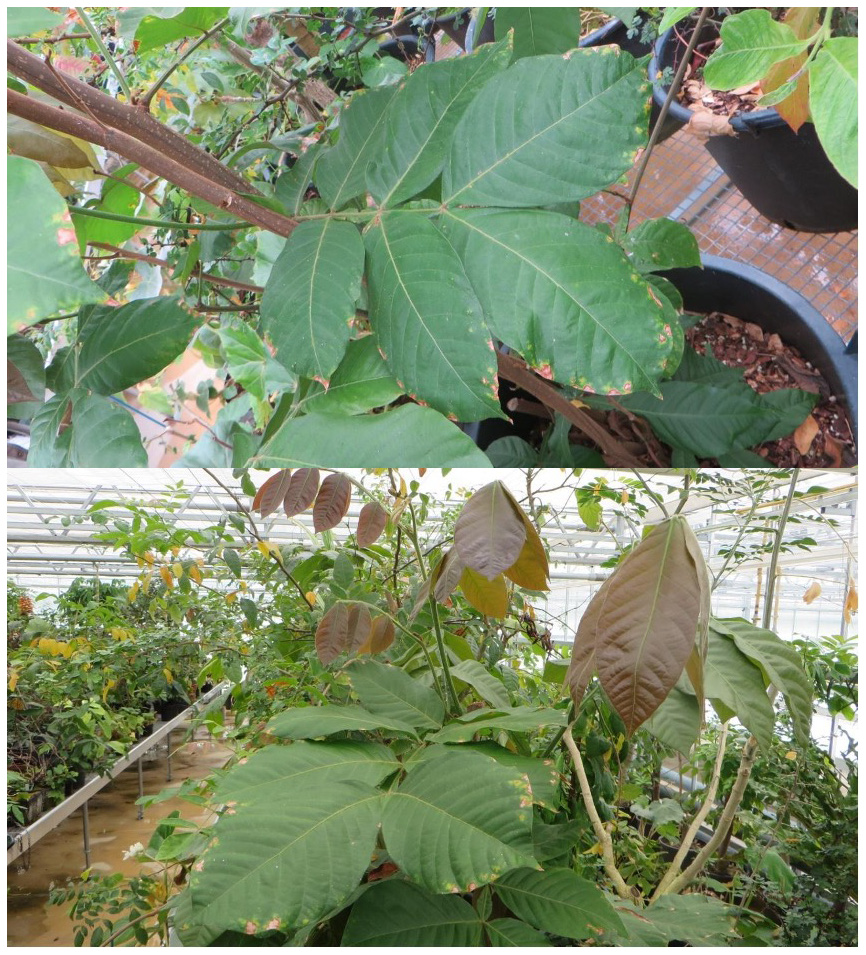
Photograph of the *Inga oerstediana* (drIngOers1) specimen used for genome sequencing collected from the living collection at Royal Botanic Garden Edinburgh, detailing mature leaves (top) and emerging young leaves (bottom).

The sequenced genome is of an *Inga oerstediana* specimen (drIngOers1, Figure 1). Using flow cytometry of leaf tissue, the genome size (1C-value) was estimated as 1.22 pg, equivalent to 1,190 Mb. The genome was sequenced using Pacific Biosciences single-molecule HiFi long reads, generating a total of 33.69 Gb (gigabases) from 2.74 million reads, providing approximately 31-fold coverage. Primary assembly contigs were scaffolded with chromosome conformation Hi-C data, which produced 101.58 Gb from 672.69 million reads, yielding an approximate coverage of 105-fold. Specimen and sequencing information is summarised in Table 1.

**Table 1.** Specimen and sequencing data for *Inga oerstediana*.

Project information			
**Study title**	Inga oerstediana		
**Umbrella BioProject**	PRJEB64756		
**Species**	*Inga oerstediana*		
**BioSample**	SAMEA111531408		
**NCBI taxonomy ID**	486073		
Specimen information			
**Technology**	**ToLID**	**BioSample accession**	**Organism part**
**PacBio long read sequencing**	drIngOers1	SAMEA111531428	Leaf
**Hi-C sequencing**	drIngOers1	SAMEA111531423	Leaf
**RNA sequencing**	drIngOers2	SAMEA113598547	Leaf
Sequencing information			
**Platform**	**Run accession**	**Read count**	**Base count (Gb)**
**Hi-C Illumina NovaSeq 6000**	ERR11814134	6.73e+08	101.58
**PacBio Sequel IIe**	ERR11809160	2.74e+06	33.69
**RNA Illumina NovaSeq 6000**	ERR12642435	6.63e+07	10.01

**Figure 2.**
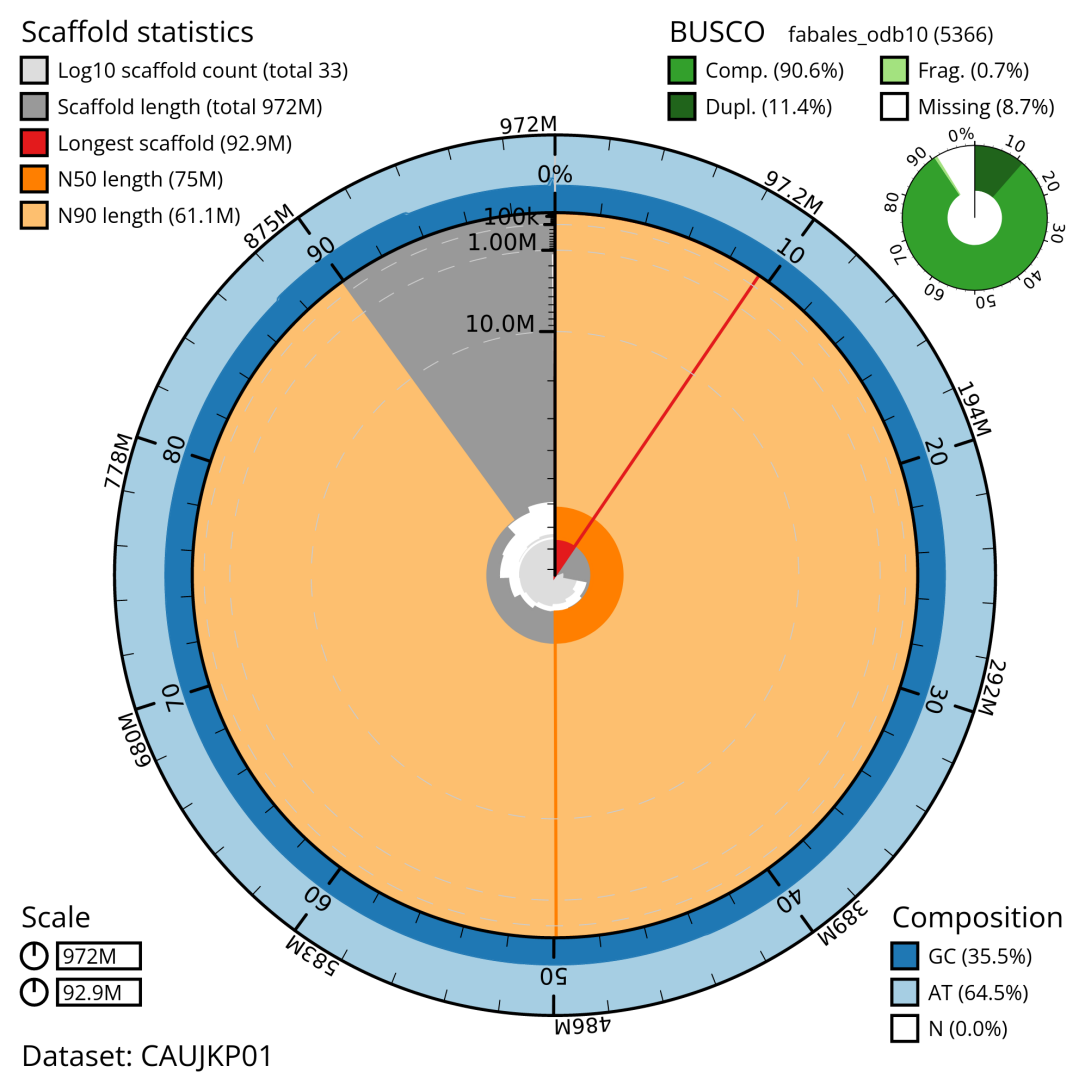
Genome assembly of *Inga oerstediana*, drIngOers1.1: metrics. The BlobToolKit snail plot shows N50 metrics and BUSCO gene completeness. The main plot is divided into 1,000 size-ordered bins around the circumference with each bin representing 0.1% of the 971,924,710 bp assembly. The distribution of scaffold lengths is shown in dark grey with the plot radius scaled to the longest scaffold present in the assembly (92,931,256 bp, shown in red). Orange and pale-orange arcs show the N50 and N90 scaffold lengths (74,992,907 and 61,111,451 bp), respectively. The pale grey spiral shows the cumulative scaffold count on a log scale with white scale lines showing successive orders of magnitude. The blue and pale-blue area around the outside of the plot shows the distribution of GC, AT and N percentages in the same bins as the inner plot. A summary of complete, fragmented, duplicated and missing BUSCO genes in the fabales_odb10 set is shown in the top right. An interactive version of this figure is available at https://blobtoolkit.genomehubs.org/view/CAUJKP01/dataset/CAUJKP01/snail.

**Figure 3.**
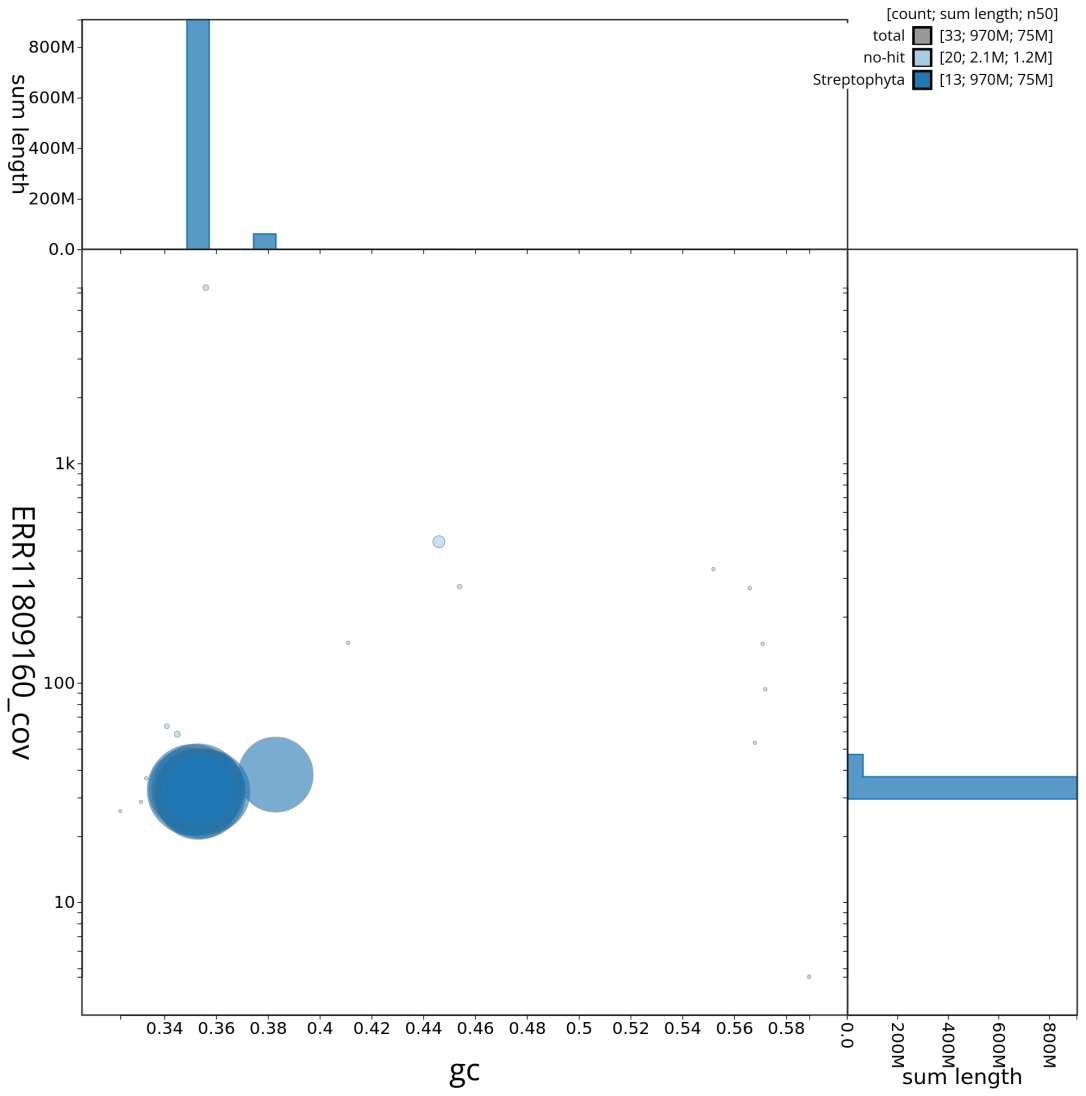
Genome assembly of *Inga oerstediana*,: Blob plot of base coverage against GC proportion for sequences in the assembly drIngOers1.1. Sequences are coloured by phylum. Circles are sized in proportion to sequence length. Histograms show the distribution of sequence length sum along each axis. An interactive version of this figure is available at https://blobtoolkit.genomehubs.org/view/CAUJKP01/dataset/CAUJKP01/blob.

**Figure 4.**
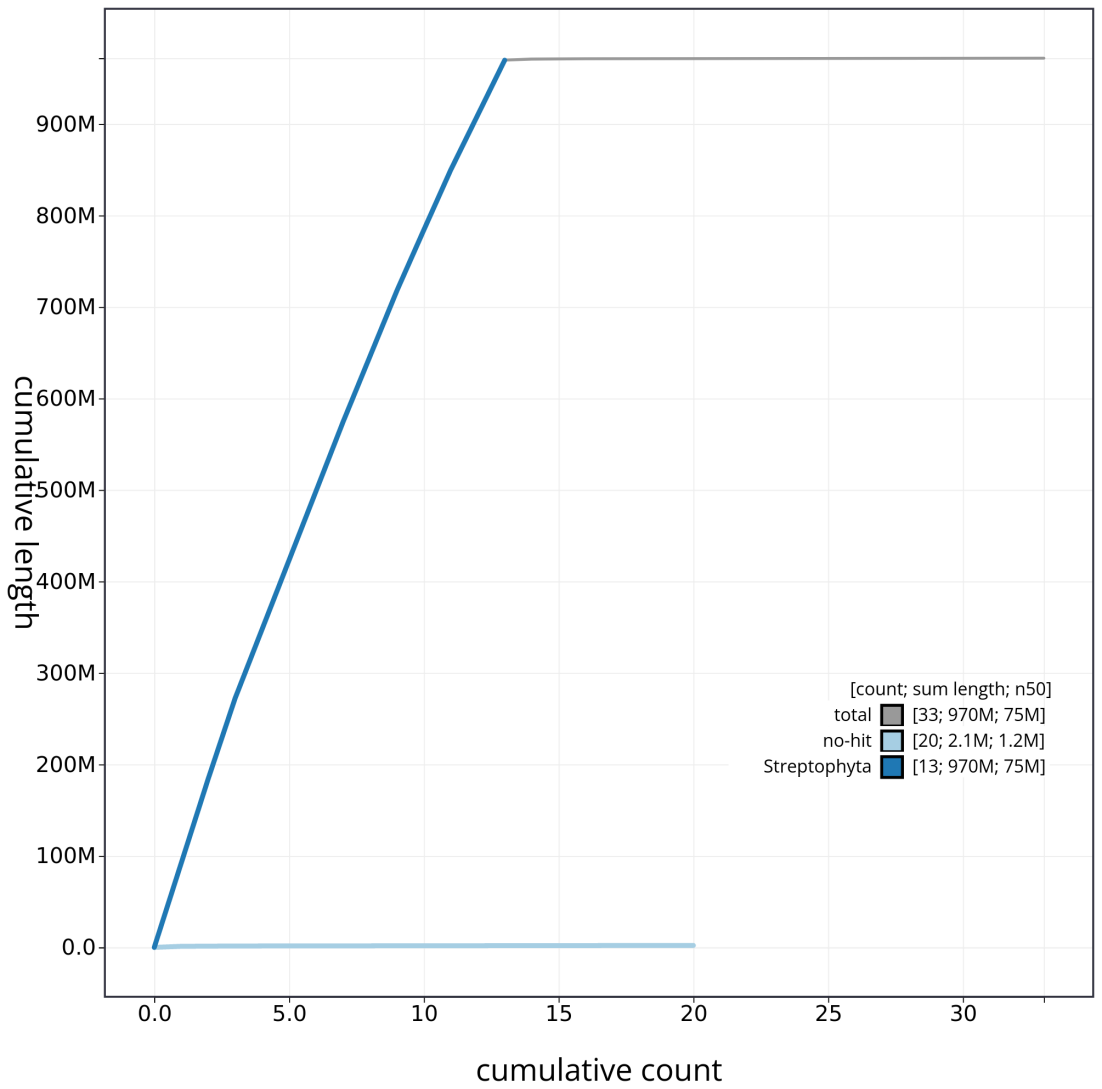
Genome assembly of *Inga oerstediana* drIngOers1.1: BlobToolKit cumulative sequence plot. The grey line shows cumulative length for all sequences. Coloured lines show cumulative lengths of sequences assigned to each phylum using the buscogenes taxrule. An interactive version of this figure is available at https://blobtoolkit.genomehubs.org/view/CAUJKP01/dataset/CAUJKP01/cumulative.

**Figure 5.**
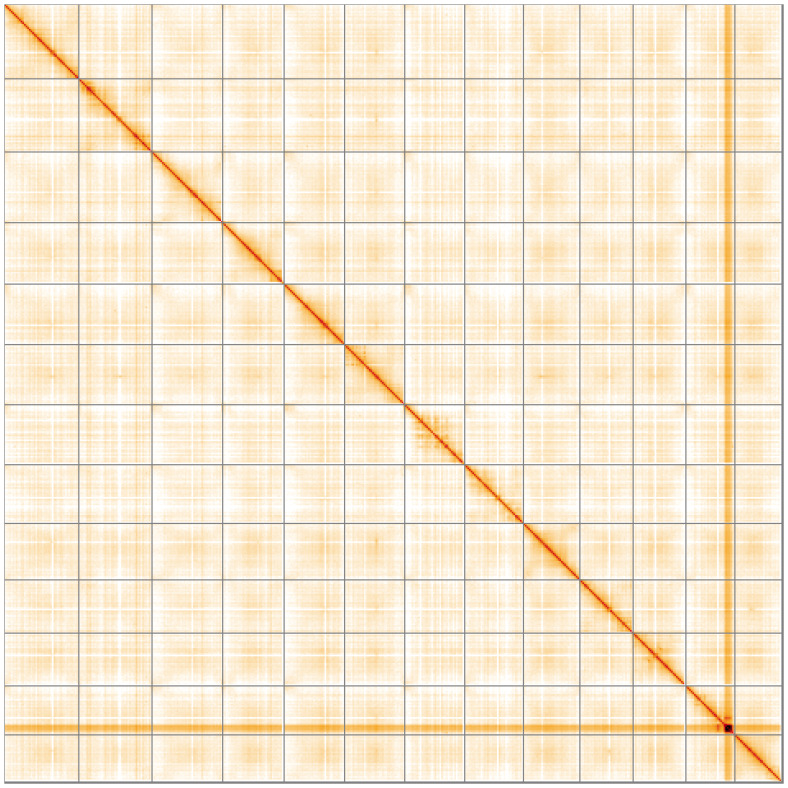
Genome assembly of *Inga oerstediana*, drIngOers1.1: Hi-C contact map of the drIngOers1.1 assembly, visualised using HiGlass. Chromosomes are shown in order of size from left to right and top to bottom. An interactive version of this figure may be viewed at https://genome-note-higlass.tol.sanger.ac.uk/l/?d=cmsVwzjASxmvp38WPTrDbw.

Manual assembly curation corrected 123 missing joins or mis-joins and 52 haplotypic duplications, reducing the assembly length by 3.38%, and decreasing the scaffold N50 by 23.22%. The final assembly has a total length of 970.60 Mb in 31 sequence scaffolds with a scaffold N50 of 75.0 Mb ( Table 2) with 312 gaps. The snail plot in Figure 2 provides a summary of the assembly statistics, while the distribution of assembly scaffolds on GC proportion and coverage is shown in Figure 3. The cumulative assembly plot in Figure 4 shows curves for subsets of scaffolds assigned to different phyla. Most (99.8%) of the assembly sequence was assigned to 13 chromosomal-level scaffolds. Chromosome-scale scaffolds confirmed by the Hi-C data are named in order of size ( Figure 5; Table 3). The order and orientation of contigs along Chromosome 12 between 49 Mb and 56 Mb is uncertain. A heterozygous inversion was observed on Chromosome 11 between 20.9 Mb and 35.3 Mb. While not fully phased, the assembly deposited is of one haplotype. Contigs corresponding to the second haplotype have also been deposited. The mitochondrial and plastid genomes were also assembled and can be found as contigs within the multifasta file of the genome submission.

**Table 2.** Genome assembly data for *Inga oerstediana*, drIngOers1.1.

Genome assembly		
Assembly name	drIngOers1.1	
Assembly accession	GCA_963210345.1	
*Accession of alternate haplotype*	*GCA_963210355.1*	
Span (Mb)	970.60	
Number of contigs	345	
Contig N50 length (Mb)	5.3	
Number of scaffolds	31	
Scaffold N50 length (Mb)	75.0	
Longest scaffold (Mb)	92.93	
Assembly metrics *		*Benchmark*
Consensus quality (QV)	64.9	*≥ 50*
*k*-mer completeness	100.0%	*≥ 95%*
BUSCO **	C:90.6%[S:79.2%,D:11.4%], F:0.7%,M:8.7%,n:5,366	*C ≥ 95%*
Percentage of assembly mapped to chromosomes	99.8%	*≥ 95%*
Organelles	Mitochondrial genome: 1166.81 kb; plastid genome: 175.18 kb	*complete single alleles*
Genome annotation at Ensembl		
Number of protein-coding genes	33,334	
Number of non-coding genes	14,645	
Number of gene transcripts	68,987	

* Assembly metric benchmarks are adapted from column VGP-2020 of “Table 1: Proposed standards and metrics for defining genome assembly quality” from Rhie *et al.* (2021). ** BUSCO scores based on the fabales_odb10 BUSCO set using version 5.4.3. C = complete [S = single copy, D = duplicated], F = fragmented, M = missing, n = number of orthologues in comparison. A full set of BUSCO scores is available at https://blobtoolkit.genomehubs.org/view/CAUJKP01/dataset/CAUJKP01/busco.

**Table 3.** Chromosomal pseudomolecules in the genome assembly of *Inga oerstediana*, drIngOers1.

INSDC accession	Name	Length (Mb)	GC%
OY723399.1	1	92.93	35.5
OY723400.1	2	91.28	35.0
OY723401.1	3	88.14	35.5
OY723402.1	4	76.57	35.5
OY723403.1	5	75.44	35.5
OY723404.1	6	74.99	35.0
OY723405.1	7	74.8	35.0
OY723406.1	8	73.21	35.5
OY723407.1	9	70.38	35.5
OY723408.1	10	66.43	35.0
OY723409.1	11	65.76	35.5
OY723410.1	12	61.11	38.5
OY723411.1	13	58.74	35.5
OY723412.1	MT	1.17	44.5
OY723413.1	Pltd	0.18	35.5

The estimated Quality Value (QV) of the final assembly is 64.9 with *k*-mer completeness of 100.0%, and the assembly has a BUSCO v5.4.3 completeness of 90.6% (single = 79.2%, duplicated = 11.4%), using the fabales_odb10 reference set ( *n* = 5,366).

Metadata for specimens, BOLD barcode results, spectra estimates, sequencing runs, contaminants and pre-curation assembly statistics are given at https://links.tol.sanger.ac.uk/species/486073.

***Genome annotation report***

The *Inga oerstediana* genome assembly (GCA_963210345.1) was annotated at the European Bioinformatics Institute (EBI) on Ensembl Rapid Release. The resulting annotation includes 68,987 transcribed mRNAs from 33,334 protein-coding and 14,645 non-coding genes ( Table 2; https://rapid.ensembl.org/Inga_oerstediana_GCA_963210345.1/Info/Index). The average transcript length is 3,483.92. There are 1.44 coding transcripts per gene and 4.80 exons per transcript.

## Methods

### Sample acquisition and nucleic acid extraction

A specimen of *Inga leiocalycina* (specimen ID SAN2000551, ToLID drIngOers1) was collected on 2021-09-09 from the wet tropics glasshouse at the Royal Botanic Garden Edinburgh, Scotland, UK. The specimen used for RNA sequencing (specimen ID SAN20001665, ToLID drIngOers2) was collected from the same individual on 2023-05-31. The specimens were collected by Rowan Schley (University of Exeter). The original individual was collected in Napo, Ecuador in 1991 and identified by Terence D. Pennington (Royal Botanic Gardens Kew). The herbarium voucher associated with the sequenced plant is RBGE:BROWP2038 and is deposited in the herbarium of the Royal Botanic Garden Edinburgh (Herbarium code: E).

The workflow for high molecular weight (HMW) DNA extraction at the Wellcome Sanger Institute (WSI) Tree of Life Core Laboratory includes a sequence of core procedures: sample preparation and homogenisation, DNA extraction, fragmentation and purification. Detailed protocols are available on protocols.io ( Denton *et al.*, 2023). The drIngOers1 sample was weighed and dissected on dry ice ( Jay *et al.*, 2023) and leaf tissue was cryogenically disrupted using the Covaris cryoPREP ^®^ Automated Dry Pulverizer ( Narváez-Gómez *et al.*, 2023).

HMW DNA was extracted using the Manual Plant MagAttract v4 protocol ( Jackson & Howard, 2023). HMW DNA was sheared into an average fragment size of 12–20 kb in a Megaruptor 3 system ( Bates *et al.*, 2023). Sheared DNA was purified by solid-phase reversible immobilisation, using AMPure PB beads to eliminate shorter fragments and concentrate the DNA ( Oatley *et al.*, 2023). The concentration of the sheared and purified DNA was assessed using a Nanodrop spectrophotometer and Qubit Fluorometer and Qubit dsDNA High Sensitivity Assay kit. Fragment size distribution was evaluated by running the sample on the FemtoPulse system.

RNA was extracted from leaf tissue of drIngOers2 in the Tree of Life Laboratory at the WSI using the RNA Extraction: Automated MagMax™ *mir*Vana protocol ( do Amaral *et al.*, 2023). The RNA concentration was assessed using a Nanodrop spectrophotometer and a Qubit Fluorometer using the Qubit RNA Broad-Range Assay kit. Analysis of the integrity of the RNA was done using the Agilent RNA 6000 Pico Kit and Eukaryotic Total RNA assay.

### Sequencing

Pacific Biosciences HiFi circular consensus DNA sequencing libraries were constructed according to the manufacturers’ instructions. Poly(A) RNA-Seq libraries were constructed using the NEB Ultra II RNA Library Prep kit. DNA and RNA sequencing was performed by the Scientific Operations core at the WSI on Pacific Biosciences Sequel IIe (HiFi) and Illumina NovaSeq 6000 (RNA-Seq) instruments. Hi-C data were also generated from leaf tissue of drIngOers1 using the Arima-HiC v2 kit. The Hi-C sequencing was performed using paired-end sequencing with a read length of 150 bp on the Illumina NovaSeq 6000 instrument.

### Genome assembly, curation and evaluation

***Assembly***

The HiFi reads were first assembled using Hifiasm ( Cheng *et al.*, 2021) with the --primary option. Haplotypic duplications were identified and removed using purge_dups ( Guan *et al.*, 2020). The Hi-C reads were mapped to the primary contigs using bwa-mem2 ( Vasimuddin *et al.*, 2019). The contigs were further scaffolded using the provided Hi-C data ( Rao *et al.*, 2014) in YaHS ( Zhou *et al.*, 2023) using the --break option. The scaffolded assemblies were evaluated using Gfastats ( Formenti *et al.*, 2022), BUSCO ( Manni *et al.*, 2021) and MERQURY.FK ( Rhie *et al.*, 2020). The organelle genomes were assembled using OATK ( Zhou, 2023).

***Curation***

The assembly was decontaminated using the Assembly Screen for Cobionts and Contaminants (ASCC) pipeline (article in preparation). Manual curation was primarily conducted using PretextView ( Harry, 2022), with additional insights provided by JBrowse2 ( Diesh *et al.*, 2023) and HiGlass ( Kerpedjiev *et al.*, 2018). Scaffolds were visually inspected and corrected as described by Howe *et al*. (2021). Any identified contamination, missed joins, and mis-joins were corrected, and duplicate sequences were tagged and removed. The process is documented at https://gitlab.com/wtsi-grit/rapid-curation (article in preparation).

***Evaluation of final assembly***

A Hi-C map for the final assembly was produced using bwa-mem2 ( Vasimuddin *et al.*, 2019) in the Cooler file format ( Abdennur & Mirny, 2020). To assess the assembly metrics, the *k*-mer completeness and QV consensus quality values were calculated in Merqury ( Rhie *et al.*, 2020). This work was done using the “sanger-tol/readmapping” ( Surana *et al.*, 2023a) and “sanger-tol/genomenote” ( Surana *et al.*, 2023b) pipelines. The genome readmapping pipelines were developed using the nf-core tooling ( Ewels *et al.*, 2020), use MultiQC ( Ewels *et al.*, 2016), and make extensive use of the Conda package manager, the Bioconda initiative ( Grüning *et al.*, 2018), the Biocontainers infrastructure ( da Veiga Leprevost *et al.*, 2017), and the Docker ( Merkel, 2014) and Singularity ( Kurtzer *et al.*, 2017) containerisation solutions. The genome was also analysed within the BlobToolKit environment ( Challis *et al.*, 2020) and BUSCO scores ( Manni *et al.*, 2021) were calculated.

Table 4 contains a list of relevant software tool versions and sources.

**Table 4.** Software tools: versions and sources.

Software tool	Version	Source
BlobToolKit	4.2.1	https://github.com/blobtoolkit/blobtoolkit
BUSCO	5.3.2	https://gitlab.com/ezlab/busco
bwa-mem2	2.2.1	https://github.com/bwa-mem2/bwa-mem2
Cooler	0.8.11	https://github.com/open2c/cooler
Gfastats	1.3.6	https://github.com/vgl-hub/gfastats
Hifiasm	0.19.5-r587	https://github.com/chhylp123/hifiasm
HiGlass	1.11.6	https://github.com/higlass/higlass
Merqury	MerquryFK	https://github.com/thegenemyers/MERQURY.FK
OATK	0.9	https://github.com/c-zhou/oatk
PretextView	0.2	https://github.com/wtsi-hpag/PretextView
purge_dups	1.2.3	https://github.com/dfguan/purge_dups
sanger-tol/genomenote	v1.0	https://github.com/sanger-tol/genomenote
sanger-tol/readmapping	1.1.0	https://github.com/sanger-tol/readmapping/tree/1.1.0
YaHS	1.1a.2	https://github.com/c-zhou/yahs

### Wellcome Sanger Institute – Legal and Governance

The materials that have contributed to this genome note have been supplied by a Darwin Tree of Life Partner. The submission of materials by a Darwin Tree of Life Partner is subject to the **‘Darwin Tree of Life Project Sampling Code of Practice’**, which can be found in full on the Darwin Tree of Life website here. By agreeing with and signing up to the Sampling Code of Practice, the Darwin Tree of Life Partner agrees they will meet the legal and ethical requirements and standards set out within this document in respect of all samples acquired for, and supplied to, the Darwin Tree of Life Project.

Further, the Wellcome Sanger Institute employs a process whereby due diligence is carried out proportionate to the nature of the materials themselves, and the circumstances under which they have been/are to be collected and provided for use. The purpose of this is to address and mitigate any potential legal and/or ethical implications of receipt and use of the materials as part of the research project, and to ensure that in doing so we align with best practice wherever possible. The overarching areas of consideration are:

•     Ethical review of provenance and sourcing of the material

•     Legality of collection, transfer and use (national and international)

Each transfer of samples is further undertaken according to a Research Collaboration Agreement or Material Transfer Agreement entered into by the Darwin Tree of Life Partner, Genome Research Limited (operating as the Wellcome Sanger Institute), and in some circumstances other Darwin Tree of Life collaborators.

## Data Availability

European Nucleotide Archive: *Inga oerstediana*. Accession number PRJEB64756; https://identifiers.org/ena.embl/PRJEB64756 ( Wellcome Sanger Institute, 2023). The genome sequence is released openly for reuse. All raw sequence data and the assembly have been deposited in INSDC databases. Raw data and assembly accession identifiers are reported in Table 1.

## Author information

Members of the Wellcome Sanger Institute Tree of Life Management, Samples and Laboratory team are listed here: https://doi.org/10.5281/zenodo.12162482.

Members of Wellcome Sanger Institute Scientific Operations: Sequencing Operations are listed here: https://doi.org/10.5281/zenodo.12165051.

Members of the Wellcome Sanger Institute Tree of Life Core Informatics team are listed here: https://doi.org/10.5281/zenodo.12160324.

Members of the Tree of Life Core Informatics collective are listed here: https://doi.org/10.5281/zenodo.12205391.
