# Supplementary material for: The genome sequence of Inga oerstediana Benth
Source: Wellcome Open Res. 2024 Oct 17;9:607. [Version 1] doi: 10.12688/wellcomeopenres.23146.1 (PMC11599804; doi:10.12688/wellcomeopenres.23146.1)
Supplement: Peer review history

# Reviewer report: Reviewer response for version 1

**DOI:** 10.21956/wellcomeopenres.25489.r110805

**Published:** 2024-12-02

**Authors:** Harpreet Kaur

**Affiliations:** 1 University of Minnesota, St. Paul, Minnesota, USA

The manuscript titled **“The genome sequence of *Inga oerstediana* Benth.”** is very well written, technically sound, and appropriate, with sufficient detail provided in every section. The Materials and Methods section is particularly well-documented, offering enough information to replicate the research. Furthermore, the data has been made publicly available and is easily accessible.

 Based on its quality and the significance of the research, I believe this article should be indexed.

**Minor Suggestions:**

**Abstract**:

- In the second line, I recommend revising the phrase to: **“The assembled genome sequence has a length…”**instead of **“The genome sequence has a length…”**. Since the genome sequence is not yet fully sequenced, this revision would be more precise.
- Similarly, in the line **“The mitochondrial and plastid genome assemblies have lengths…”**, I suggest adding the word **“assembled”** for clarity.

**Introduction**:

- I suggest replacing **“chromosomally complete”** in the sentence **“Here we present one of three chromosomally complete, annotated genome sequences for *Inga*, which are the first for the genus.”** with **“chromosome-level”**.
- Additionally, the sentence is somewhat unclear. If this genome sequence is one of three, what exactly is being referred to as “the first for the genus”? Please clarify or rephrase.
- If there are previous genome sequences available for this species, consider citing them for context and comparison.

**Materials and Methods**:

 This section is exceptionally well-written and detailed. However, I have one question:

- What type of leaf tissue (young or mature) was specifically used for RNA extractions? Including this detail would improve the clarity and reproducibility of the methodology.

Are sufficient details of methods and materials provided to allow replication by others?

Yes

Is the rationale for creating the dataset(s) clearly described?

Yes

Are the datasets clearly presented in a useable and accessible format?

Yes

Are the protocols appropriate and is the work technically sound?

Yes

Reviewer Expertise:

Alfalfa Breeding and Genomics

I confirm that I have read this submission and believe that I have an appropriate level of expertise to confirm that it is of an acceptable scientific standard.

# Reviewer report: Reviewer response for version 1

**DOI:** 10.21956/wellcomeopenres.25489.r107182

**Published:** 2024-11-26

**Authors:** Liangsheng Zhang

**Affiliations:** 1 Genomics and Genetic Engineering Laboratory of Ornamental Plants, College of Agriculture and Biotechnology, Zhejiang University, Hangzhou, Zhejiang, China

*Inga oerstediana *belongs to the legume family and is an important species, and this genome deserves to be resolved. The study reported a high-quality genome, a high-quality annotated nuclear genome, a chloroplast, and a mitochondrial genome from *Inga oerstediana*. The data of the article is also publicly available, the quality is very good, and it is recommended to indexing.

Are sufficient details of methods and materials provided to allow replication by others?

Yes

Is the rationale for creating the dataset(s) clearly described?

Yes

Are the datasets clearly presented in a useable and accessible format?

Yes

Are the protocols appropriate and is the work technically sound?

Yes

Reviewer Expertise:

Plant genome and bioinformatics

I confirm that I have read this submission and believe that I have an appropriate level of expertise to confirm that it is of an acceptable scientific standard.

# Reviewer report: Reviewer response for version 1

**DOI:** 10.21956/wellcomeopenres.25489.r108041

**Published:** 2024-11-19

**Authors:** Chris Cullis

**Affiliations:** 1 Case Western Reserve University, Cleveland, Ohio, USA

This is a standard report of the assembly of a complete genome for a new species. However, there are a few pieces of information that could be addressed. The total length of the assembly was 970 Mbp, but the genome size estimate was 1190 Mbp. What is the source of this discrepancy. Are there contigs, such as the long arrays of ribosomal RNA repeats and/or 5S RNA repeats that have not assembled completely, and do they provide additional unassembled contigs? The BUSCO completeness against the fabales gene set is only 90%which is below the expected level. What genes are missing, and are they consistent with expectations for a species with this range? It is noted in passing that the mitochondrial and chloroplast genomes were also assembled. Since both of these organellar genomes are usually present in different structural forms due to intermolecular recombination between repeats, is there evidence for these or did all the reads unambiguously align with the assemblies?

Are sufficient details of methods and materials provided to allow replication by others?

Yes

Is the rationale for creating the dataset(s) clearly described?

Yes

Are the datasets clearly presented in a useable and accessible format?

Yes

Are the protocols appropriate and is the work technically sound?

Yes

Reviewer Expertise:

Plant genome characterization

I confirm that I have read this submission and believe that I have an appropriate level of expertise to confirm that it is of an acceptable scientific standard.
